# Poly[di-μ_3_-hy­droxy[μ_4_-5-(4-carb­oxy­phen­yl)pyridine-2-carboxyl­ato-κ^5^ *N*,*O* ^2^:*O* ^2′^:*O* ^4^:*O* ^4′^]dicadmium]

**DOI:** 10.1107/S1600536812042444

**Published:** 2012-10-13

**Authors:** Fan-Jin Meng, Heng-Qing Jia, Ning-Hai Hu, Hua Zhou

**Affiliations:** a State Key Laboratory of Electroanalytical Chemistry, Changchun Institute of Applied Chemistry, Chinese Academy of Sciences, Changchun 130022, People’s Republic of China

## Abstract

The asymmetric unit of the title polymeric complex, [Cd_2_(C_13_H_7_NO_4_)(OH)_2_]_*n*_, consists of two independent Cd^II^ atoms, one 5-(4-carb­oxy­phen­yl)pyridine-2-carboxyl­ate ligand and two hy­droxy groups. One Cd^II^ atom is six-coordinated by two O atoms from two ligand mol­ecules and by four μ_3_-OH groups in a distorted trigonal–prismatic geometry. The other is five-coordinated by one N and two O atoms from two ligands and by two μ_3_-OH groups, forming a distorted square-pyramidal geometry. The two independent Cd^II^ atoms are connected by the ligand mol­ecules and the OH groups into a three-dimensional framework. O—H⋯O hydrogen bonds between the OH groups and the carboxyl­ate O atoms are observed.

## Related literature

For related structures and applications of metal complexes with *N*-heterocyclic multicarboxyl­ate ligands, see: Li *et al.* (2008 ▶); Mahata & Natarajan (2005 ▶); Sun *et al.* (2001 ▶); Wang *et al.* (2009 ▶). For the synthesis of the ligand, see: Ben & Gordon (1951 ▶); Liu *et al.* (2005 ▶).
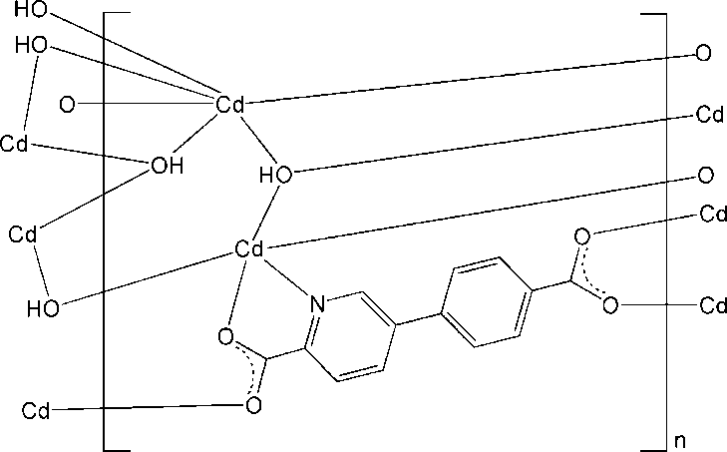


## Experimental

### 

#### Crystal data

[Cd_2_(C_13_H_7_NO_4_)(OH)_2_]
*M* *_r_* = 500.03
Monoclinic, 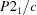

*a* = 15.4316 (17) Å
*b* = 3.8261 (4) Å
*c* = 21.586 (2) Å
β = 102.114 (2)°
*V* = 1246.1 (2) Å^3^
*Z* = 4
Mo *K*α radiation
μ = 3.44 mm^−1^
*T* = 293 K
0.27 × 0.21 × 0.14 mm

#### Data collection

Bruker APEX CCD diffractometer
Absorption correction: multi-scan (*SADABS*; Bruker, 2001 ▶) *T*_min_ = 0.457, *T*_max_ = 0.644
6277 measured reflections
2448 independent reflections
2109 reflections with *I* > 2σ(*I*)
*R*_int_ = 0.052

#### Refinement

*R*[*F* ^2^ > 2σ(*F* ^2^)] = 0.037
*wR*(*F* ^2^) = 0.087
*S* = 1.04
2448 reflections
205 parameters
2 restraints
H atoms treated by a mixture of independent and constrained refinement
Δρ_max_ = 1.82 e Å^−3^
Δρ_min_ = −1.36 e Å^−3^

### 

Data collection: *SMART* (Bruker, 2007 ▶); cell refinement: *SAINT* (Bruker, 2007 ▶); data reduction: *SAINT*; program(s) used to solve structure: *SHELXS97* (Sheldrick, 2008 ▶); program(s) used to refine structure: *SHELXL97* (Sheldrick, 2008 ▶); molecular graphics: *SHELXTL* (Sheldrick, 2008 ▶); software used to prepare material for publication: *SHELXTL*.

## Supplementary Material

Crystal structure: contains datablock(s) global, I. DOI: 10.1107/S1600536812042444/is5190sup1.cif

Structure factors: contains datablock(s) I. DOI: 10.1107/S1600536812042444/is5190Isup2.hkl

Additional supplementary materials:  crystallographic information; 3D view; checkCIF report

## Figures and Tables

**Table 1:** Hydrogen-bond geometry (Å, °)

*D*—H⋯*A*	*D*—H	H⋯*A*	*D*⋯*A*	*D*—H⋯*A*
O5—H5*A*⋯O1^i^	0.81 (3)	2.08 (4)	2.818 (6)	150 (6)
O6—H6*A*⋯O3^ii^	0.82 (2)	2.39 (4)	2.887 (6)	120 (6)

Symmetry codes: (i) 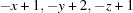; (ii) 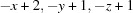.

**Fig. 1.**
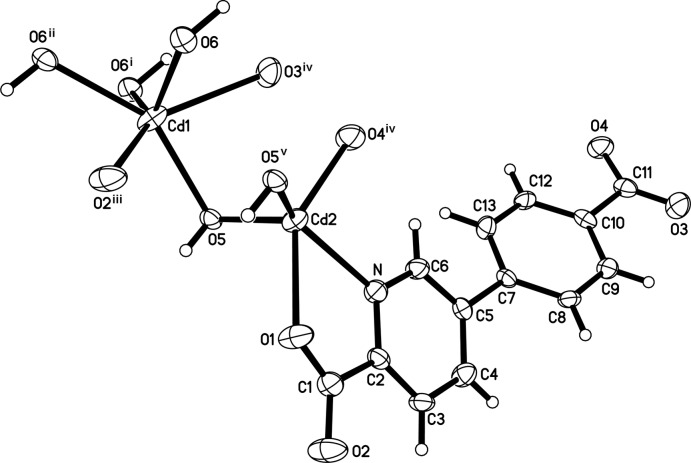
The asymmetric unit of the title compound. Displacement ellipsoids are drawn at the 50% probability level. [Symmetry codes: (i) x, -1+y, z; (ii) 1-x, -1/2+y, 1/2-z; (iii) 1-x, 2-y, 1-z; (iv) 2-x, 1-y, 1-z; (v) x, 1+y, z.]

**Fig. 2.**
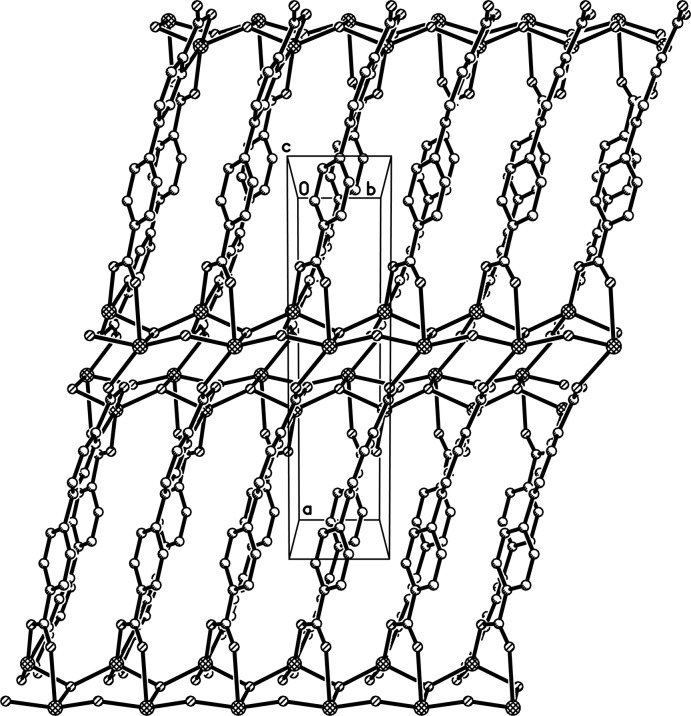
The packing diagram of the title compound, showing the three-dimensional framework.

## supplementary crystallographic information

### Comment

The rational design and construction of coordination polymers based on metal ions and N-heterocyclic multicarboxylate ligands have attracted considerable attention for their intriguing structural topologies along with potential applications (Li *et al.*, 2008; Mahata & Natarajan, 2005; Sun *et al.*, 2001; Wang *et al.*, 2009). In this paper, we report a cadmium complex with a three-dimensional framework based on 5-(4-carboxyphenyl)pyridine-2-carboxylic acid (H_2_*L*).

The asymmetric unit of the title compound contains two crystallographically independent Cd^II^ ions with different coordination geometries (Fig. 1). The Cd1 atom is six-coordinated by two O atoms from two *L* ligands and four µ_3_-OH groups in a distorted trigonal prismatic geometry. The Cd1—O bond lengths are in a range of 2.176 (4)–2.587 (4) Å. The Cd2 atom is five-coordinated by one N and two O atoms from two *L* ligands and two µ_3_-OH groups in a distorted square-pyramidal geometry. The Cd2—N bond length is 2.354 (4) Å and the Cd2—O bond lengths are in a range of 2.196 (4)–2.368 (4) Å. Interestingly, the carboxylate groups and hydroxy groups connect the Cd^II^ ions into a layer parallel to (011) and adjacent layers are further linked by the *L* ligands as pillars along the *a*-axis, generating a three-dimensional framework (Fig. 2). O—H···O hydrogen bonds between the hydroxy groups and carboxylate O atoms stabilize the structure (Table 1).

### Experimental

The H_2_*L* ligand was prepared by a similar method described by Ben & Gordon (1951) and Liu *et al.* (2005). A precursor ligand, 2-methyl-5-p-tolylpyridine, was prepared through the Suzuki reaction between 4-methylphenylboronic acid (2.039 g, 15 mmol) and 5-bromo-2-methylpyridine (1.730 g, 10 mmol). The H_2_*L* ligand was obtained by the oxidation of potassium permanganate. The title compound was synthesized under hydrothermal conditions. A mixture of Cd(CH_3_CO_2_)_2_.2H_2_O (0.053 g, 0.2mmol) and H_2_*L* (0.024 g, 0.1 mmol) in methanol (2 ml) and distilled water (5 ml) was stirred for 20 min in air, and the pH value was adjusted to about 8.5 with 0.1M CH_3_COOH and 0.1M NaOH solutions. Then the mixture was sealed in a 23 ml Teflon-lined stainless steel autoclave, which was heated to 433 K for 72 h. After cooling to room temperature, colorless block crystals of the title compound suitable for X-ray diffraction were obtained.

### Refinement

C-bound H atoms were positioned geometrically and refined as riding atoms, with C—H = 0.93 Å and with *U*_iso_(H) = 1.2*U*_eq_(C). H atoms of hydroxy groups were located in a difference Fourier map and their positions were refined with bond-length restraints of 0.82 (1) Å, and with *U*_iso_(H) = 1.5*U*_eq_(O). The highest residual electron density was found at 0.96 Å from Cd1 atom and the deepest hole at 0.70 Å from Cd1 atom.

### Crystal data

**Table:**

[Cd_2_(C_13_H_7_NO_4_)(OH)_2_]	*F*(000) = 952
*M**_r_* = 500.03	*D*_x_ = 2.665 Mg m^−^^3^
Monoclinic, *P*2_1_/*c*	Mo *K*α radiation, λ = 0.71073 Å
Hall symbol: -P 2ybc	Cell parameters from 2448 reflections
*a* = 15.4316 (17) Å	θ = 2.1–26.0°
*b* = 3.8261 (4) Å	µ = 3.44 mm^−^^1^
*c* = 21.586 (2) Å	*T* = 293 K
β = 102.114 (2)°	Block, colourless
*V* = 1246.1 (2) Å^3^	0.27 × 0.21 × 0.14 mm
*Z* = 4	

### Data collection

**Table:**

Bruker APEX CCD diffractometer	2448 independent reflections
Radiation source: fine-focus sealed tube	2109 reflections with *I* > 2σ(*I*)
Graphite monochromator	*R*_int_ = 0.052
φ and ω scans	θ_max_ = 26.0°, θ_min_ = 2.1°
Absorption correction: multi-scan (*SADABS*; Bruker, 2001)	*h* = −19→14
*T*_min_ = 0.457, *T*_max_ = 0.644	*k* = −3→4
6277 measured reflections	*l* = −26→26

### Refinement

**Table:**

Refinement on *F*^2^	Primary atom site location: structure-invariant direct methods
Least-squares matrix: full	Secondary atom site location: difference Fourier map
*R*[*F*^2^ > 2σ(*F*^2^)] = 0.037	Hydrogen site location: inferred from neighbouring sites
*wR*(*F*^2^) = 0.087	H atoms treated by a mixture of independent and constrained refinement
*S* = 1.04	*w* = 1/[σ^2^(*F*_o_^2^) + (0.0384*P*)^2^ + 3.8222*P*] where *P* = (*F*_o_^2^ + 2*F*_c_^2^)/3
2448 reflections	(Δ/σ)_max_ = 0.001
205 parameters	Δρ_max_ = 1.82 e Å^−^^3^
2 restraints	Δρ_min_ = −1.36 e Å^−^^3^

### Special details

**Table:**

Geometry. All esds (except the esd in the dihedral angle between two l.s. planes) are estimated using the full covariance matrix. The cell esds are taken into account individually in the estimation of esds in distances, angles and torsion angles; correlations between esds in cell parameters are only used when they are defined by crystal symmetry. An approximate (isotropic) treatment of cell esds is used for estimating esds involving l.s. planes.
Refinement. Refinement of F^2^ against ALL reflections. The weighted R-factor wR and goodness of fit S are based on F^2^, conventional R-factors R are based on F, with F set to zero for negative F^2^. The threshold expression of F^2^ > 2sigma(F^2^) is used only for calculating R-factors(gt) etc. and is not relevant to the choice of reflections for refinement. R-factors based on F^2^ are statistically about twice as large as those based on F, and R- factors based on ALL data will be even larger.

### Fractional atomic coordinates and isotropic or equivalent isotropic displacement parameters (Å^2^)

**Table:**

	*x*	*y*	*z*	*U*_iso_*/*U*_eq_	
Cd1	0.54015 (3)	0.60259 (12)	0.317372 (18)	0.02773 (14)	
Cd2	0.63702 (3)	1.00568 (11)	0.464037 (18)	0.02162 (14)	
C1	0.6046 (4)	1.0297 (15)	0.6045 (3)	0.0230 (12)	
C2	0.6959 (3)	0.8660 (15)	0.6131 (3)	0.0197 (11)	
C3	0.7430 (3)	0.7675 (16)	0.6720 (3)	0.0226 (12)	
H3	0.7181	0.7837	0.7075	0.027*	
C4	0.8292 (4)	0.6427 (16)	0.6769 (3)	0.0251 (13)	
H4	0.8629	0.5804	0.7163	0.030*	
C5	0.8646 (3)	0.6115 (14)	0.6239 (3)	0.0185 (11)	
C6	0.8105 (3)	0.7034 (16)	0.5659 (3)	0.0231 (12)	
H6	0.8327	0.6758	0.5293	0.028*	
C7	0.9584 (3)	0.5045 (14)	0.6255 (3)	0.0185 (11)	
C8	1.0268 (3)	0.6001 (15)	0.6757 (2)	0.0216 (12)	
H8	1.0139	0.7186	0.7103	0.026*	
C9	1.1138 (3)	0.5190 (15)	0.6742 (3)	0.0216 (12)	
H9	1.1589	0.5883	0.7076	0.026*	
C10	1.1350 (3)	0.3358 (15)	0.6238 (3)	0.0216 (12)	
C11	1.2310 (4)	0.2519 (16)	0.6218 (3)	0.0241 (12)	
C12	1.0658 (3)	0.2371 (15)	0.5748 (2)	0.0224 (12)	
H12	1.0782	0.1098	0.5410	0.027*	
C13	0.9795 (4)	0.3229 (16)	0.5751 (3)	0.0238 (13)	
H13	0.9347	0.2583	0.5412	0.029*	
N1	0.7283 (3)	0.8293 (13)	0.5604 (2)	0.0224 (10)	
O1	0.5713 (3)	1.1503 (12)	0.55044 (18)	0.0330 (10)	
O2	0.5712 (3)	1.0362 (13)	0.6518 (2)	0.0408 (12)	
O3	1.2893 (3)	0.3539 (12)	0.66625 (19)	0.0342 (10)	
O4	1.2417 (3)	0.0784 (12)	0.57409 (19)	0.0324 (10)	
O5	0.5786 (2)	0.5013 (10)	0.42462 (18)	0.0197 (8)	
H5A	0.5284 (17)	0.528 (16)	0.430 (3)	0.030*	
O6	0.5682 (2)	1.1069 (11)	0.27639 (19)	0.0238 (8)	
H6A	0.6206 (13)	1.093 (18)	0.275 (3)	0.036*	

### Atomic displacement parameters (Å^2^)

**Table:**

	*U*^11^	*U*^22^	*U*^33^	*U*^12^	*U*^13^	*U*^23^
Cd1	0.0398 (3)	0.0251 (3)	0.0162 (2)	0.00757 (19)	0.00127 (18)	−0.00045 (18)
Cd2	0.0247 (2)	0.0217 (2)	0.0182 (2)	0.00551 (16)	0.00379 (16)	−0.00076 (16)
C1	0.020 (3)	0.025 (3)	0.025 (3)	0.006 (2)	0.006 (2)	−0.001 (2)
C2	0.017 (3)	0.021 (3)	0.023 (3)	−0.001 (2)	0.008 (2)	−0.002 (2)
C3	0.021 (3)	0.031 (3)	0.018 (3)	0.003 (2)	0.009 (2)	0.003 (2)
C4	0.025 (3)	0.029 (3)	0.020 (3)	0.005 (2)	0.002 (2)	0.000 (3)
C5	0.016 (3)	0.016 (3)	0.024 (3)	−0.001 (2)	0.005 (2)	0.000 (2)
C6	0.019 (3)	0.033 (3)	0.018 (3)	0.004 (2)	0.005 (2)	0.000 (3)
C7	0.014 (2)	0.022 (3)	0.021 (3)	−0.001 (2)	0.007 (2)	0.000 (2)
C8	0.022 (3)	0.030 (3)	0.015 (2)	0.001 (2)	0.007 (2)	0.000 (2)
C9	0.018 (3)	0.027 (3)	0.019 (3)	0.003 (2)	0.001 (2)	0.004 (2)
C10	0.020 (3)	0.021 (3)	0.026 (3)	0.001 (2)	0.010 (2)	0.007 (2)
C11	0.023 (3)	0.027 (3)	0.024 (3)	0.003 (2)	0.008 (2)	0.008 (3)
C12	0.023 (3)	0.029 (3)	0.018 (3)	0.001 (2)	0.008 (2)	−0.006 (3)
C13	0.020 (3)	0.028 (3)	0.022 (3)	−0.001 (2)	−0.001 (2)	0.001 (2)
N1	0.018 (2)	0.028 (3)	0.022 (2)	0.0040 (19)	0.0048 (19)	−0.002 (2)
O1	0.027 (2)	0.053 (3)	0.020 (2)	0.022 (2)	0.0073 (17)	0.008 (2)
O2	0.035 (2)	0.064 (3)	0.027 (2)	0.025 (2)	0.015 (2)	0.010 (2)
O3	0.022 (2)	0.049 (3)	0.031 (2)	0.006 (2)	0.0018 (18)	−0.003 (2)
O4	0.028 (2)	0.046 (3)	0.026 (2)	0.006 (2)	0.0105 (18)	−0.004 (2)
O5	0.0139 (17)	0.025 (2)	0.0215 (19)	0.0029 (15)	0.0058 (15)	0.0022 (16)
O6	0.0217 (19)	0.023 (2)	0.028 (2)	0.0027 (17)	0.0088 (17)	−0.0046 (18)

### Geometric parameters (Å, º)

**Table:**

Cd1—O6^i^	2.174 (4)	C6—H6	0.9300
Cd1—O6	2.203 (4)	C7—C13	1.384 (8)
Cd1—O5	2.299 (4)	C7—C8	1.393 (7)
Cd1—O6^ii^	2.339 (4)	C8—C9	1.385 (7)
Cd1—O2^iii^	2.405 (4)	C8—H8	0.9300
Cd1—O3^iv^	2.586 (4)	C9—C10	1.391 (8)
Cd2—O5^v^	2.193 (4)	C9—H9	0.9300
Cd2—O4^iv^	2.221 (4)	C10—C12	1.388 (7)
Cd2—O5	2.222 (4)	C10—C11	1.525 (7)
Cd2—N1	2.354 (4)	C11—O3	1.233 (7)
Cd2—O1	2.368 (4)	C11—O4	1.264 (7)
C1—O2	1.237 (7)	C12—C13	1.373 (7)
C1—O1	1.261 (7)	C12—H12	0.9300
C1—C2	1.517 (7)	C13—H13	0.9300
C2—N1	1.342 (7)	O2—Cd1^iii^	2.405 (4)
C2—C3	1.377 (7)	O3—Cd1^iv^	2.586 (4)
C3—C4	1.396 (7)	O4—Cd2^iv^	2.221 (4)
C3—H3	0.9300	O5—Cd2^i^	2.193 (4)
C4—C5	1.372 (8)	O5—H5A	0.814 (10)
C4—H4	0.9300	O6—Cd1^v^	2.174 (4)
C5—C6	1.396 (7)	O6—Cd1^vi^	2.339 (4)
C5—C7	1.498 (7)	O6—H6A	0.817 (10)
C6—N1	1.339 (7)		
			
O6^i^—Cd1—O6	121.88 (18)	C5—C6—H6	118.4
O6^i^—Cd1—O5	103.38 (14)	C13—C7—C8	118.5 (5)
O6—Cd1—O5	121.41 (14)	C13—C7—C5	120.3 (5)
O6^i^—Cd1—O6^ii^	79.70 (13)	C8—C7—C5	121.1 (5)
O6—Cd1—O6^ii^	79.12 (13)	C9—C8—C7	120.2 (5)
O5—Cd1—O6^ii^	149.21 (13)	C9—C8—H8	119.9
O6^i^—Cd1—O2^iii^	145.94 (16)	C7—C8—H8	119.9
O6—Cd1—O2^iii^	79.60 (15)	C8—C9—C10	121.3 (5)
O5—Cd1—O2^iii^	82.34 (13)	C8—C9—H9	119.4
O6^ii^—Cd1—O2^iii^	79.22 (14)	C10—C9—H9	119.4
O6^i^—Cd1—O3^iv^	80.11 (14)	C12—C10—C9	117.6 (5)
O6—Cd1—O3^iv^	73.66 (14)	C12—C10—C11	121.2 (5)
O5—Cd1—O3^iv^	80.39 (13)	C9—C10—C11	121.2 (5)
O6^ii^—Cd1—O3^iv^	129.78 (13)	O3—C11—O4	127.0 (5)
O2^iii^—Cd1—O3^iv^	133.69 (16)	O3—C11—C10	117.7 (5)
O5^v^—Cd2—O4^iv^	106.95 (15)	O4—C11—C10	115.3 (5)
O5^v^—Cd2—O5	120.10 (16)	C13—C12—C10	121.5 (5)
O4^iv^—Cd2—O5	92.19 (15)	C13—C12—H12	119.2
O5^v^—Cd2—N1	135.39 (16)	C10—C12—H12	119.2
O4^iv^—Cd2—N1	83.77 (15)	C12—C13—C7	120.8 (5)
O5—Cd2—N1	102.06 (15)	C12—C13—H13	119.6
O5^v^—Cd2—O1	83.94 (14)	C7—C13—H13	119.6
O4^iv^—Cd2—O1	149.23 (14)	C6—N1—C2	118.5 (5)
O5—Cd2—O1	107.35 (16)	C6—N1—Cd2	124.1 (4)
N1—Cd2—O1	69.28 (14)	C2—N1—Cd2	117.3 (3)
O2—C1—O1	126.7 (5)	C1—O1—Cd2	119.1 (3)
O2—C1—C2	116.5 (5)	C1—O2—Cd1^iii^	133.3 (4)
O1—C1—C2	116.8 (5)	C11—O3—Cd1^iv^	133.0 (4)
N1—C2—C3	122.3 (5)	C11—O4—Cd2^iv^	129.9 (4)
N1—C2—C1	116.2 (5)	Cd2^i^—O5—Cd2	120.10 (16)
C3—C2—C1	121.5 (5)	Cd2^i^—O5—Cd1	122.32 (17)
C2—C3—C4	118.3 (5)	Cd2—O5—Cd1	103.73 (15)
C2—C3—H3	120.9	Cd2^i^—O5—H5A	112 (5)
C4—C3—H3	120.9	Cd2—O5—H5A	99 (5)
C5—C4—C3	120.4 (5)	Cd1—O5—H5A	94 (4)
C5—C4—H4	119.8	Cd1^v^—O6—Cd1	121.87 (18)
C3—C4—H4	119.8	Cd1^v^—O6—Cd1^vi^	101.02 (15)
C4—C5—C6	117.2 (5)	Cd1—O6—Cd1^vi^	100.16 (15)
C4—C5—C7	123.8 (5)	Cd1^v^—O6—H6A	111 (5)
C6—C5—C7	118.8 (5)	Cd1—O6—H6A	104 (5)
N1—C6—C5	123.1 (5)	Cd1^vi^—O6—H6A	120 (5)
N1—C6—H6	118.4		
			
O2—C1—C2—N1	173.9 (6)	O1—Cd2—N1—C2	5.9 (4)
O1—C1—C2—N1	−7.3 (8)	O2—C1—O1—Cd2	−168.3 (5)
O2—C1—C2—C3	−7.3 (8)	C2—C1—O1—Cd2	13.0 (7)
O1—C1—C2—C3	171.5 (6)	O5^v^—Cd2—O1—C1	−154.0 (5)
N1—C2—C3—C4	3.5 (9)	O4^iv^—Cd2—O1—C1	−40.8 (6)
C1—C2—C3—C4	−175.3 (5)	O5—Cd2—O1—C1	86.3 (5)
C2—C3—C4—C5	−1.7 (9)	N1—Cd2—O1—C1	−10.4 (4)
C3—C4—C5—C6	−1.1 (9)	O1—C1—O2—Cd1^iii^	−22.3 (10)
C3—C4—C5—C7	175.5 (5)	C2—C1—O2—Cd1^iii^	156.4 (4)
C4—C5—C6—N1	2.3 (9)	O4—C11—O3—Cd1^iv^	13.6 (10)
C7—C5—C6—N1	−174.4 (5)	C10—C11—O3—Cd1^iv^	−167.4 (4)
C4—C5—C7—C13	149.3 (6)	O3—C11—O4—Cd2^iv^	−17.1 (9)
C6—C5—C7—C13	−34.2 (8)	C10—C11—O4—Cd2^iv^	163.9 (4)
C4—C5—C7—C8	−34.0 (8)	O5^v^—Cd2—O5—Cd2^i^	−179.992 (1)
C6—C5—C7—C8	142.5 (6)	O4^iv^—Cd2—O5—Cd2^i^	69.0 (2)
C13—C7—C8—C9	1.1 (8)	N1—Cd2—O5—Cd2^i^	−15.1 (2)
C5—C7—C8—C9	−175.6 (5)	O1—Cd2—O5—Cd2^i^	−86.94 (19)
C7—C8—C9—C10	−1.3 (9)	O5^v^—Cd2—O5—Cd1	39.0 (3)
C8—C9—C10—C12	0.0 (8)	O4^iv^—Cd2—O5—Cd1	−72.10 (16)
C8—C9—C10—C11	179.0 (5)	N1—Cd2—O5—Cd1	−156.20 (15)
C12—C10—C11—O3	178.5 (6)	O1—Cd2—O5—Cd1	132.00 (14)
C9—C10—C11—O3	−0.4 (8)	O6^i^—Cd1—O5—Cd2^i^	3.5 (2)
C12—C10—C11—O4	−2.4 (8)	O6—Cd1—O5—Cd2^i^	−137.88 (18)
C9—C10—C11—O4	178.7 (5)	O6^ii^—Cd1—O5—Cd2^i^	95.8 (3)
C9—C10—C12—C13	1.4 (9)	O2^iii^—Cd1—O5—Cd2^i^	149.3 (2)
C11—C10—C12—C13	−177.6 (5)	O3^iv^—Cd1—O5—Cd2^i^	−73.8 (2)
C10—C12—C13—C7	−1.6 (9)	O6^i^—Cd1—O5—Cd2	143.42 (15)
C8—C7—C13—C12	0.3 (9)	O6—Cd1—O5—Cd2	2.1 (2)
C5—C7—C13—C12	177.1 (5)	O6^ii^—Cd1—O5—Cd2	−124.3 (2)
C5—C6—N1—C2	−0.7 (9)	O2^iii^—Cd1—O5—Cd2	−70.76 (17)
C5—C6—N1—Cd2	177.7 (4)	O3^iv^—Cd1—O5—Cd2	66.10 (15)
C3—C2—N1—C6	−2.3 (9)	O6^i^—Cd1—O6—Cd1^v^	180.0
C1—C2—N1—C6	176.5 (5)	O5—Cd1—O6—Cd1^v^	−45.7 (2)
C3—C2—N1—Cd2	179.2 (4)	O6^ii^—Cd1—O6—Cd1^v^	109.5 (2)
C1—C2—N1—Cd2	−2.0 (6)	O2^iii^—Cd1—O6—Cd1^v^	28.6 (2)
O5^v^—Cd2—N1—C6	−115.5 (5)	O3^iv^—Cd1—O6—Cd1^v^	−113.2 (2)
O4^iv^—Cd2—N1—C6	−7.7 (5)	O6^i^—Cd1—O6—Cd1^vi^	70.1 (2)
O5—Cd2—N1—C6	83.3 (5)	O5—Cd1—O6—Cd1^vi^	−155.55 (12)
O1—Cd2—N1—C6	−172.6 (5)	O6^ii^—Cd1—O6—Cd1^vi^	−0.37 (9)
O5^v^—Cd2—N1—C2	62.9 (5)	O2^iii^—Cd1—O6—Cd1^vi^	−81.26 (15)
O4^iv^—Cd2—N1—C2	170.8 (4)	O3^iv^—Cd1—O6—Cd1^vi^	136.96 (17)
O5—Cd2—N1—C2	−98.3 (4)		

Symmetry codes: (i) *x*, *y*−1, *z*; (ii) −*x*+1, *y*−1/2, −*z*+1/2; (iii) −*x*+1, −*y*+2, −*z*+1; (iv) −*x*+2, −*y*+1, −*z*+1; (v) *x*, *y*+1, *z*; (vi) −*x*+1, *y*+1/2, −*z*+1/2.

### Hydrogen-bond geometry (Å, º)

**Table:**

*D*—H···*A*	*D*—H	H···*A*	*D*···*A*	*D*—H···*A*
O5—H5*A*···O1^iii^	0.81 (3)	2.08 (4)	2.818 (6)	150 (6)
O6—H6*A*···O3^iv^	0.82 (2)	2.39 (4)	2.887 (6)	120 (6)

Symmetry codes: (iii) −*x*+1, −*y*+2, −*z*+1; (iv) −*x*+2, −*y*+1, −*z*+1.
